# Cryo-EM reveals a previously unrecognized structural protein of a dsRNA virus implicated in its extracellular transmission

**DOI:** 10.1371/journal.ppat.1009396

**Published:** 2021-03-17

**Authors:** Qianqian Shao, Xudong Jia, Yuanzhu Gao, Zhe Liu, Huan Zhang, Qiqi Tan, Xin Zhang, Huiqiong Zhou, Yinyin Li, De Wu, Qinfen Zhang

**Affiliations:** 1 State Key Lab for Biocontrol, School of Life Sciences, Sun Yat-sen University, Guangzhou, China; 2 Guangdong Provincial Center for Disease Control and Prevention, Guangdong Provincial Institute of Public Health, Guangzhou, China; Purdue University, UNITED STATES

## Abstract

Mosquito viruses cause unpredictable outbreaks of disease. Recently, several unassigned viruses isolated from mosquitoes, including the Omono River virus (OmRV), were identified as totivirus-like viruses, with features similar to those of the Totiviridae family. Most reported members of this family infect fungi or protozoans and lack an extracellular life cycle stage. Here, we identified a new strain of OmRV and determined high-resolution structures for this virus using single-particle cryo-electron microscopy. The structures feature an unexpected protrusion at the five-fold vertex of the capsid. Disassociation of the protrusion could result in several conformational changes in the major capsid. All these structures, together with some biological results, suggest the protrusions’ associations with the extracellular transmission of OmRV.

## Author summary

Mosquito is a reservoir of viruses, with a large amount of them perform significant research value. Omono River virus (OmRV) has been isolated from *Culex* mosquito and is closely related to the family Totiviridae. However, current researches have reported the extracellular transmission ability of OmRV, which is lacked in most members of Totiviridae. In the current study, we isolated a new strain, OmRV-LZ, and obtained its high-resolution cryo-electron microscopy (cryo-EM) structure. Unexpectedly, a protrusion structure has been found located at the five-fold vertex, which is unrecognized in the previous studies. Structural and molecular biological experiments were applied to try to investigate its functions. The results may be helpful to understand the extracellular transmission ability of OmRV-LZ and similar double-stranded RNA (dsRNA) viruses.

## Introduction

Mosquitoes carry a variety of viruses, some of which spread only among mosquitoes (mosquito-specific), while others are transmitted from mosquitoes to humans or other hosts (mosquito-borne), causing severe diseases of hosts (*e*.*g*., dengue virus) [1,2]. Some mosquito-specific viruses are thought to be ancestors of some mosquito-borne viruses [3], while some others appear to interfere with the replication and transmission of medically significant viruses, such as West Nile virus [4,5] and dengue virus [6]. Therefore, mosquito-associated viruses, whether mosquito-specific or mosquito-borne, are a matter of widespread public concern. In recent years, several novel unassigned mosquito-specific viruses, such as Omono River virus (OmRV) [7], or mosquito-borne viruses, such as *Armigeres subalbatus* totivirus (AsTV) [8], were identified as being related to the viral family Totiviridae based on phylogenetic analyses and temporarily nominated as “totivirus-like viruses”. Traditionally, members of Totiviridae infect either fungi or a number of medically important protozoan parasites, such as Trichomonas, Leishmania and Giardia [9–13], and most of them are incapable of extracellular transmission; rather, they are transmitted vertically through cell division or cell fusion [10–13]. The only exception is *Giardia lamblia virus* (GLV), which can infect the host extracellularly [9,14]. However, several “totivirus-like viruses” (including the mosquito viruses OmRV and AsTV mentioned above) have been found to be infectious in metazoan cell cultures or individual organisms, suggesting that they are capable of extracellular transmission [7,8,15–17].

Infectious myonecrosis virus (IMNV), the first isolated totivirus-like virus, has been identified as the infectious agent that led to the collapse of shrimp yields in Brazil [15,16]. Notably, an 8 Å cryo-EM structure of IMNV revealed a fibre-like protrusion, which was considered to mediate cell recognition/binding and entry [18]. However, the biochemistry and structural basis of these extracellular transmission pathways are still poorly understood. Generally, members of Totiviridae and totivirus-like viruses have a non-segmented double-stranded RNA (dsRNA) genome containing two large overlapping open reading frames (ORFs), namely, ORF1 and ORF2, which encode the major capsid protein (MCP) and RNA-dependent RNA polymerase (RdRP), respectively [7]. However, in both cases of IMNV and OmRV, which have shown to be capable of extracellular transmission, ORF1 encodes a poly-protein precursor [7,15]. The N-termini of the IMNV and OmRV MCPs are generated by cleavage after a conserved oligopeptide sequence motif “NKxMHxxNGN” [7,15]. Furthermore, upstream of this cleavage site, ORF1 has two “2A-like” cleavage motifs, resulting in three additional small peptide fragments: a putative dsRNA-binding protein (dsRBP) containing a dsRNA-binding motif and two other unidentified peptides [19,20]. It is believed that at least one of these peptides forms a fibre-like protrusion in IMNV [18]. Nevertheless, the previously reported OmRV non-protruding structures raise further questions regarding the poorly understood protrusion structure and extracellular transmission mechanism of totivirus-like viruses [21,22].

Here, we report cryo-EM structures of a newly isolated OmRV strain (named OmRV-LZ). The results show that OmRV-LZ harbours unexpected pentamer protrusions and reveal that the protrusion is composed of a small protein cleaved from the polypeptide precursor encoded by ORF1. We further demonstrated that the protrusion might associate with the extracellular transmission of OmRV-LZ.

## Results

### Virus isolation and identification

*Culex* samples were collected from Leizhou, Guangdong Province (PR of China). After homogenization and centrifugation, the supernatants of the samples were added to C6/36 cells and could have serious cytopathic effects (CPEs) on these cells. Sample was then amplified and purified by gradient ultra-centrifugation for subsequent analyses.

Next-generation sequencing and *de novo* contig assembly revealed a genome sequence of 7613 nucleotides (nt). A BLASTn search revealed that the sequence most closely matched that of OmRV-AK4 (7611 nt; GenBank code AB555544.1) [7] with 97% homology. Accordingly, we designated the isolated virus as a new strain of OmRV and called it OmRV-LZ.

**Fig 1:**
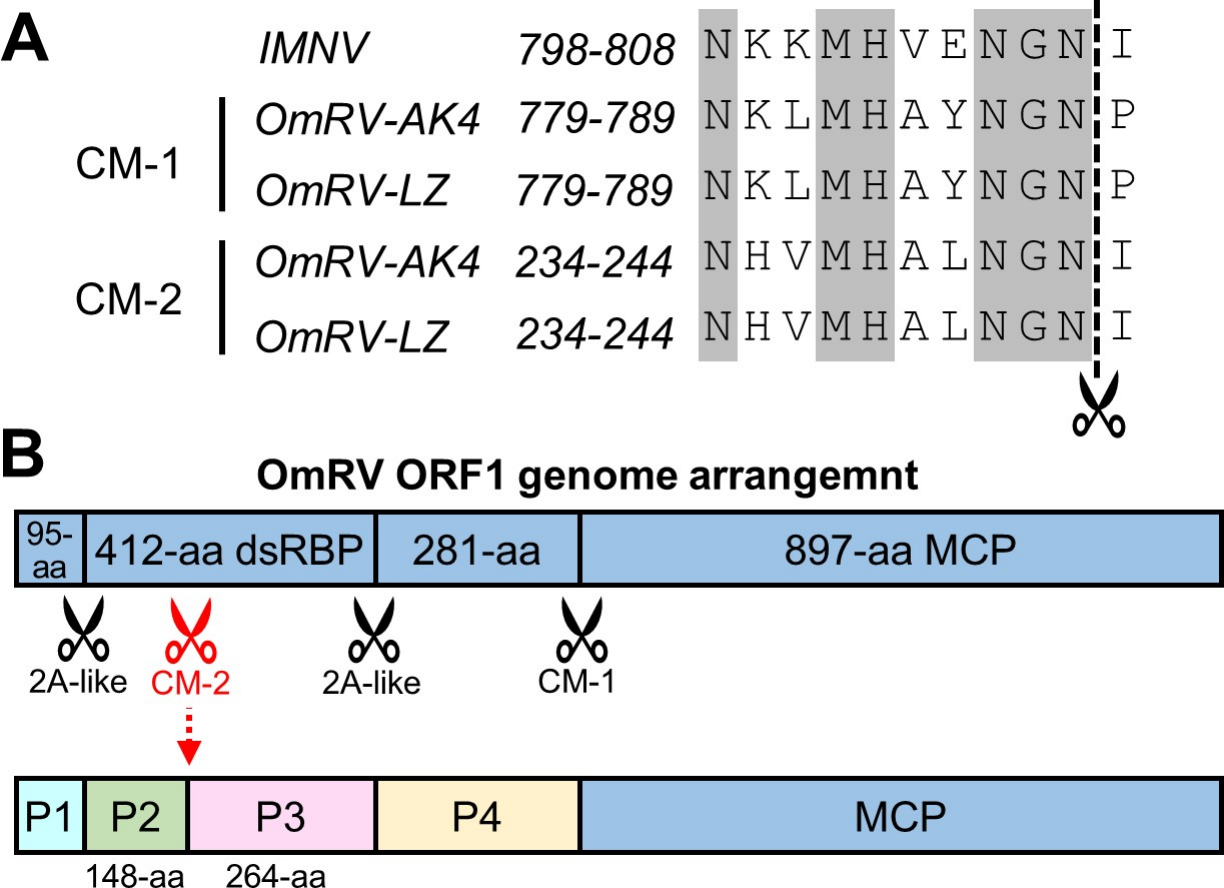
Genome characterization of OmRV-LZ. **(A)** Amino acid sequence alignment of a typical cleavage site motif in OmRV and IMNV. The dotted line indicates the cleavage site. GenBank accession numbers: IMNV, AY570982; OmRV-AK4, AB555544; OmRV-LZ, MT066059. **(B)** Genome arrangement of OmRV-LZ ORF1. The previously reported cleavage sites are indicated by black scissor logos. A newly found cleavage site (CM-2, indicated by red scissor logo) resulted in dsRBP being cleaved into two fragments with lengths of 148 and 264 amino acids, as shown. The resulting four small protein fragments are named P1, P2, P3 and P4.

It has been demonstrated that the MCPs of OmRV and IMNV were generated from a precursor peptide [7,15]. The 10 residues upstream of the cleavage site are highly conserved between OmRV (residues 779–788) and IMNV (residues 798–807). We temporarily named this conserved 10-residue peptide segment as the cleavage motif (CM). Interestingly, in the ORF1 of both OmRV-LZ and OmRV-AK4, we found another 10 amino acids (10-aa) segment, ‘NHVMHALNGN’ (residues 234–243), that is highly similar to the motif (Fig 1A). It is reasonable to infer that the site between residues Asn243 and Ile244 may also be a cleavage site. For simplicity, we designated these putative cleavage site motifs in OmRV as CM-1 (residues 779–788) and CM-2 (residues 234–243). Cleavage at putative CM-2 site would result in the dsRBP being further cleaved into a 148-aa (approximately 16.6 kD) fragment and a 264-aa (approximately 28.0 kD) fragment. Together with the previously reported 2A-like cleavage, the precursor peptide should be cleaved to 95-, 148-, 264- and 281-aa fragments named P1 to P4, respectively, from the N- to C-terminus if CM-2 truly exists (Fig 1B). Herein, it is unclear whether OmRV-LZ has protrusions and which fragment forms the protrusion if there is a protrusion. To answer these questions, single-particle cryo-EM was further applied to study the structure of OmRV-LZ.

### Overall structure of OmRV-LZ

**Fig 2:**
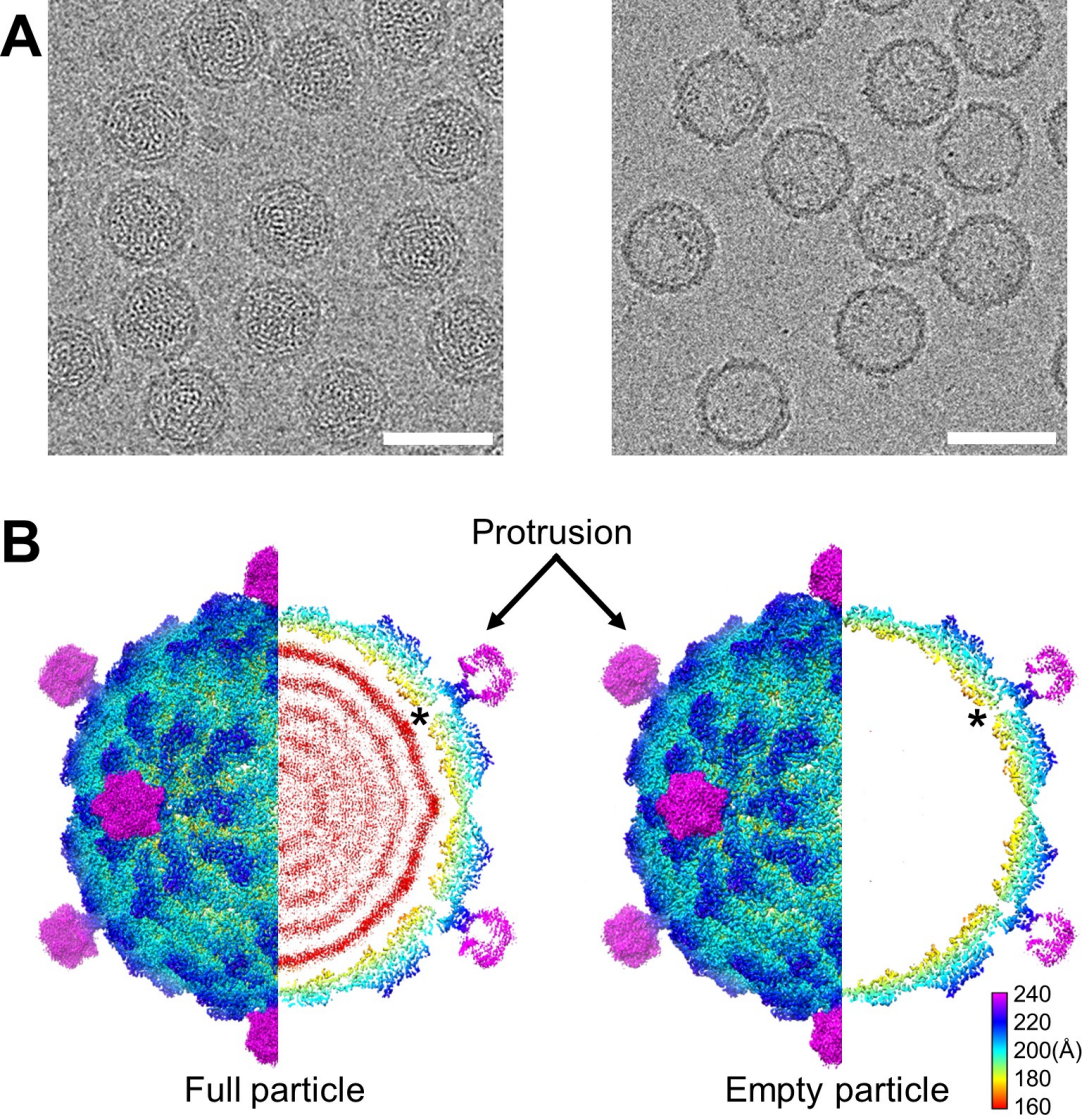
Overall structures of OmRV-LZ. **(A)** Representative Cryo-EM micrographs of OmRV-LZ samples with genome (left) and without genome (right). Scale bar = 50nm. **(B)** Surface view and cross-section of 3-D maps of full (left) and empty (right) OmRV-LZ particles. Maps are coloured radially. The black asterisk indicates the pore at the five-fold vertex. To show each element of the virus, maps are shown at different counter levels. The major capsids of the full and empty particles are displayed at 4.0σ and 2.9σ, respectively; sub-volumes of the protrusion of the full and empty particles are displayed at 0.65σ and 0.97σ, respectively; nucleic acid layers of the full particle are displayed at 1.5σ.

Two fractions were obtained after CsCl density gradient ultra-centrifugation, and both of the fragments were subjected to further structure determination. The cryo-EM micrographs and two-dimensional (2-D) class averaging revealed that the lower and upper fractions from ultra-centrifugation contained virus particles with and without the genome, respectively (Fig 2A). The final two three-dimensional (3-D) maps were resolved at 2.79 Å (full particles with genome) and 3.40 Å (empty particles without genome), respectively, based on the Fourier shell correlation (FSC) 0.143 criterion (S1A Fig). Upon comparing these two maps, we found that these two structures were identical except for the genome (Fig 2B). Unexpectedly, both 3-D maps revealed that OmRV-LZ had obvious protrusions at the five-fold vertexes (Fig 2B), which is different from the previously reported structures of OmRV-AK4, which has no protrusion [21,22].

The diameter of the OmRV-LZ without the protrusions is 450 Å, and that with protrusions is 540 Å. Similar to that of other members of Totiviridae, OmRV-LZ has a major capsid with icosahedral symmetry and a T = 1 lattice comprising 60 dimers composed of MCP-A and MCP-B. The central cross-sectional view of the map with the genome shows at least five visible layers of viral genome RNA packaged inside the capsid (Fig 2B). A pore with an average diameter of ~10 Å is discernible at each five-fold vertex. Coincidentally, the protrusions are located upon the pores, almost blocking them (Fig 2B). The local resolution of the protrusion is lower than that of the MCP, suggesting that the protrusion is flexible.

### Structural features of major capsid protein

The current resolution of the map for the full particle allows us to build *ab initio* atomic models of the MCPs (S1B Fig).

**Fig 3:**
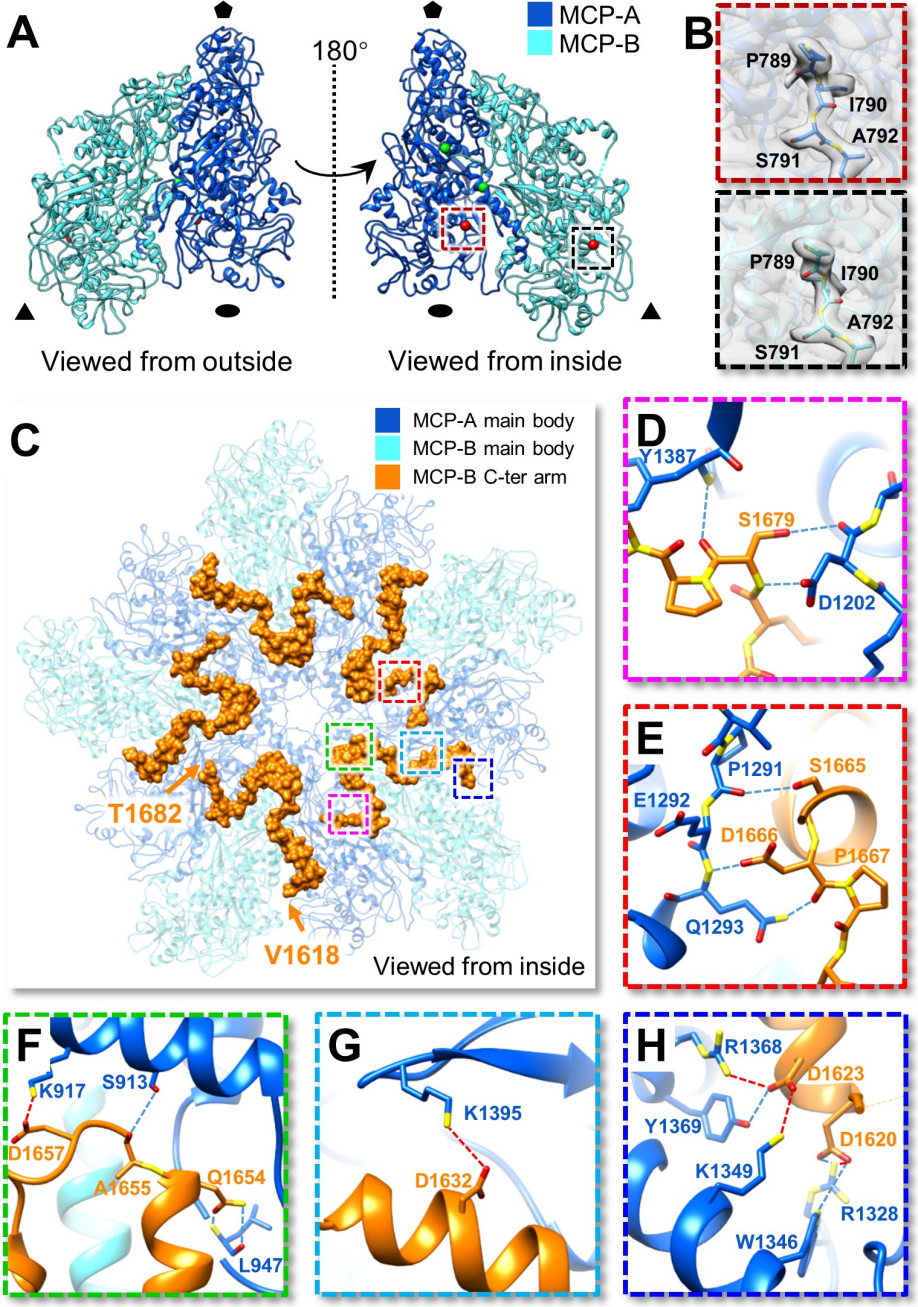
Atomic models of MCP-A and MCP-B. **(A)** Asymmetric unit of OmRV-LZ. MCP-A and MCP-B are coloured blue and cyan, respectively. Black pentagons, ovals and triangles indicate the five-fold, two-fold and three-fold axes, respectively. Red and green spheres indicate the visible N-terminus and C-terminus, respectively. **(B)** Zoomed-in views of the boxed region in panel A indicate the N-terminus of MCP-A and MCP-B. The first four residues of both MCP-A and MCP-B are shown as atomic models superposed with their densities. **(C)** The MCP decamer around the five-fold vertex is fastened by the “Ω-like” C-terminal arms of MCP-B (residues Val1618 to Thr1682, highlighted by the orange surface). **(D-H)** Zoomed-in views of the boxed areas in panel C. **(D, E)** Interactions between the MCP-B C-terminus and MCP-A within the same asymmetric unit. **(F-H)** Interactions between the MCP-B C-terminus and MCP-A in the neighbouring asymmetry unit. Hydrogen bonds and salt bridges are indicated by blue and red dotted lines, respectively.

In each asymmetric unit, MCP-A and MCP-B share a similar tertiary structure (Fig 3A), and the overall structures of these two subunits are well aligned with each other, showing a root mean square deviation (RMSD) of 0.66 Å for 728 Cα atom pairs. Each MCP-A is located close to the five-fold vertex, while MCP-B fills the vicinity of the three-fold axis. The *ab initio* models revealed that the first residue of the N-terminus of both MCP-A and MCP-B was clearly located at Pro789 (Fig 3B), which provides strong evidence for cleavage at CM-1 between residues Asn788 and Pro789 in OmRV.

Local conformational changes between MCP-A and B occur at several loops, located at residues 827–839, 920–933, 1048–1056, 1291–1302, 1357–1365, 1375–1389, 1451–1458, 1471–1477 and 1484–1489, which are almost all situated at the contact surfaces between these two subunits. Despite these local conformational changes, the most striking difference was observed at the C-terminal region. The C-terminal residues 1641–1685 of MCP-A are disordered. However, the C-terminus of MCP-B is well organized, except for residues 1611–1617 and the last three residues, 1683–1685. The MCP-B C-terminal residues 1618–1682 form a Ω-like arm structure composed of four loops and three α-helices (Fig 3C). It extends along the interface between MCP A-B-A and interacts extensively with two neighbouring MCP-As (Fig 3C). First, residues Ser1665, Asp1666, Pro1667, and Ser1679 of MCP-B and residues Asp1202, Pro1291, Glu1292, Gln1293, and Tyr1387 of MCP-A are involved in the formation of six hydrogen bonds within the same asymmetry unit (Fig 3D and 3E). Then, residues Asp1620, Asp1623, Asp1632, Gln1654, Ala1655, and Asp1657 of MCP-B and residues Ser913, Lys917, Leu947, Arg1328, Trp1346, Lys 1349, Arg1368, and Tyr1369 of MCP-A in neighbouring asymmetry units contribute to six hydrogen bonds and five salt bridges (Fig 3F–3H). All these MCP A-B-A interactions strongly fasten the capsid decamer around the five-fold axis (Fig 3C).

The structure of *Saccharomyces cerevisiae* virus L-A (ScV-L-A), another member of Totiviridae, has been determined by X-ray crystallography [23]. To escape from the RNA degradation mechanism of the host cell and improve the expression level, ScV-L-A transfers the 5’ cap structure 7-methyl-GMP (m7Gp) from the host mRNA to the newly transcribed viral RNA, forming a functional 5’ cap structure [24]. To investigate whether OmRV-LZ also possesses this functional structure, we tried to align the OmRV-LZ MCP-A to the MCP-A of ScV-L-A (S2A Fig). The results revealed that the MCP-As of OmRV-LZ and ScV-L-A share similar folds and have a conserved helix-rich core containing five pairs of α-helices (S2A and S2B Fig), although no amino sequence similarity was observed. The cap-snatching active centre of ScV-L-A is located at the outer surface of the capsid. In the active centre, four loops form a trench, and the active site, His154, is located at the tip of the trench (S2C Fig) [23,25]. However, no obvious trench structure and no histidine in OmRV-LZ could be found around the corresponding area of the cap-snatching active centre (S2D Fig).

### Annotation and structural features of the protrusion

To date, only two structures of totivirus-like viruses, OmRV-AK4 and IMNV, have been determined, and only IMNV has been reported to have a fibre-like protrusion [18,21,22]. However, the resolution of the IMNV 3-D map is too low to interpret the structural details of the protrusion. Currently, no information could ensure which peptide constitutes the protrusion.

**Fig 4:**
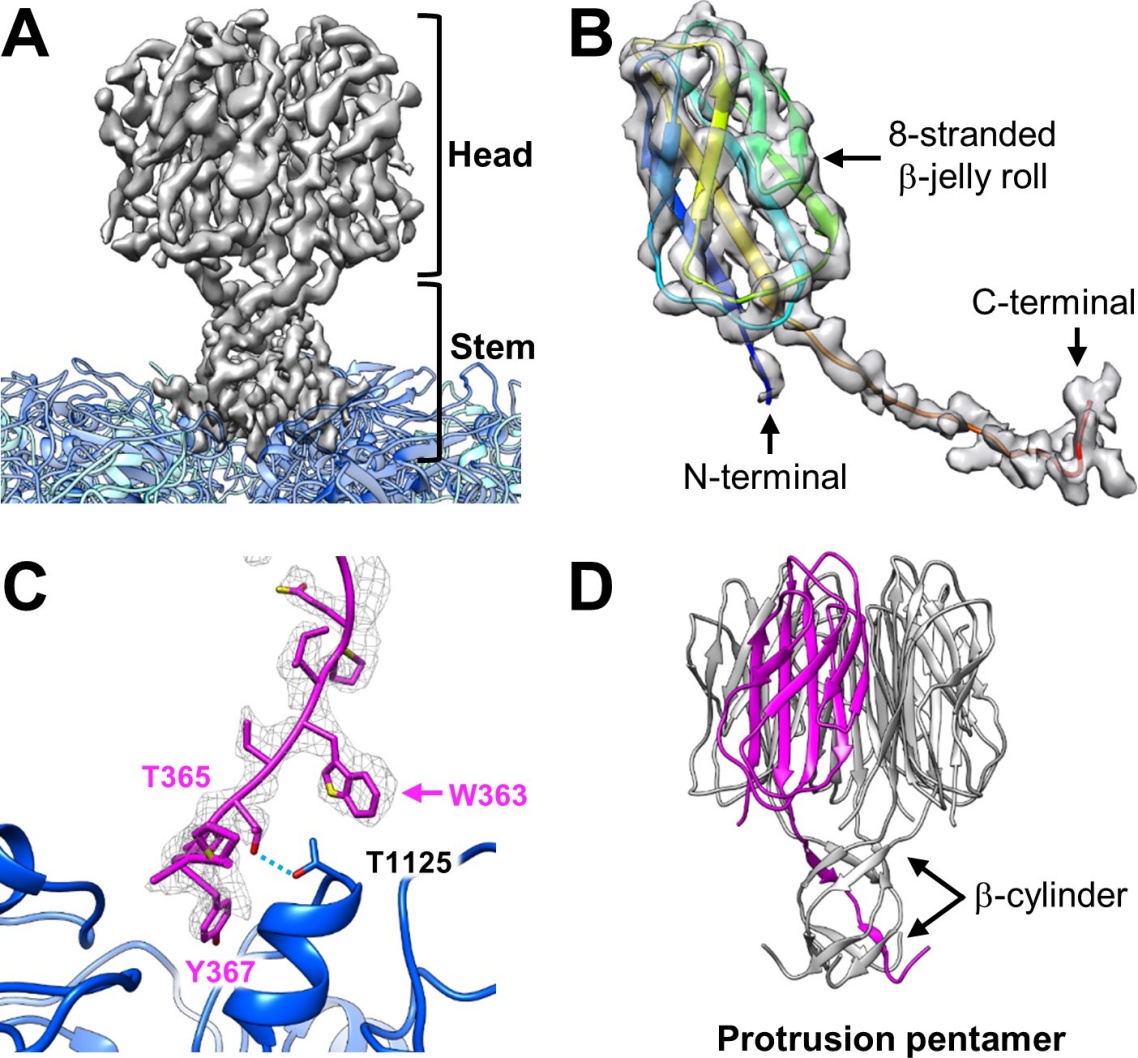
Structure of the OmRV-LZ protrusion. **(A)** Cryo-EM density maps of the protrusion. The protrusion can be divided into domain heads and stems. **(B)** The alanine backbone model of the protrusion superposed with its density map shows a β-jelly roll structure containing eight β-strands. The ribbon model is rainbow coloured from blue (N-terminal) to red (C-terminal). **(C)** Atomic model of the C-terminus of the protrusion (magenta) reveals that the sidechains fit quite well into the density map. The typical tryptophan-like sidechain density, used as the “landmark” during model building, is indicated by a magenta arrow. Two MCP-A models holding one protrusion monomer are shown as ribbons in blue. The hydrogen bond between the protrusion Thr365 and MCP-A Thr1125 is indicated by a light blue dotted line. **(D)** Side view of the full atomic model of the protrusion pentamer with one monomer coloured magenta and others coloured grey. Black arrows indicate two β-cylinder structures.

To obtain better resolution and avoid the artificial features resulting from the imposed icosahedral symmetry, we applied sub-particle refinement with C1 symmetry to the protrusion. The result shows that the protrusion of OmRV-LZ is exactly pentameric; C5 symmetry was then applied to the final reconstruction of the protrusion. The final resolution of the protrusion is 4.10 Å (S1A Fig). Overall, the protrusion can be roughly divided into two parts: a head and a stem (Fig 4A). The head has a width of ~60 Å and a height of ~40 Å. The stem is ~30 Å in both length and width. Each head is linked to the capsid via a stem, which inserts the capsid inward by approximately 10 Å (Fig 4A).

The resolution of the protrusion is good enough to build a rough poly-alanine backbone model containing 126 alanines (Fig 4B). The backbone model reveals that the protrusion head is composed of a β-jelly roll including eight β-strands in each subunit (Fig 4B).

On the other hand, the quality of the stem structure is good enough to clearly identify several large sidechains of the residues (Fig 4C). A significant tryptophan-like sidechain density could be served as a ‘landmark’ to conduct the assignment of the remaining residues (Fig 4C). There are 11 tryptophans in residues 1–788. Among them, only residue Trp363 and the adjacent residues could match the stem densities very well (Fig 4C). Finally, residues Ile244 to Pro369 belonging to P3 were assigned to the protrusion. Excitingly, the traced first residue of the N-terminus, Ile244, is just located behind the CM-2 motif, which means that the N-terminus of the protrusion is released from the ORF1-encoded precursor.

The full atomic model of the protrusion pentamer reveals a channel throughout the middle of the protrusion (S3A Fig). This channel is negatively charged at the top opening, with a diameter of ~8 Å, while the widest space has a diameter of ~18 Å, forming a cavity with a volume of ~4.9×10^3^ Å^3^ (S3A Fig). The C-terminal residues Pro349 to Pro369 compose the stem, where two β-cylinder structures are formed (Fig 4D). These two β-cylinder structures are composed of five separate peptide chains, promoting the stability of the protrusion pentamer. The C-terminus of the stem interacts with the tip of the MCP-A, whereas there is no direct interaction between the protrusion and the MCP-B. Only one hydrogen bond was found between Thr365 of each protrusion subunit and Thr1125 of MCP-A, and no salt bridge could be identified (Fig 4C). The sidechain of Tyr367 is inserted into a positively charged canyon, which is formed by two adjacent MCP-As (Figs 4C and S3B).

### Structure of OmRV-LZ without the protrusion

Among all 2-D classes, those without protrusion can be found in both full (S4 Fig) and empty particles. The proportion of protrusion-free particles in empty ones is very lower.

**Fig 5:**
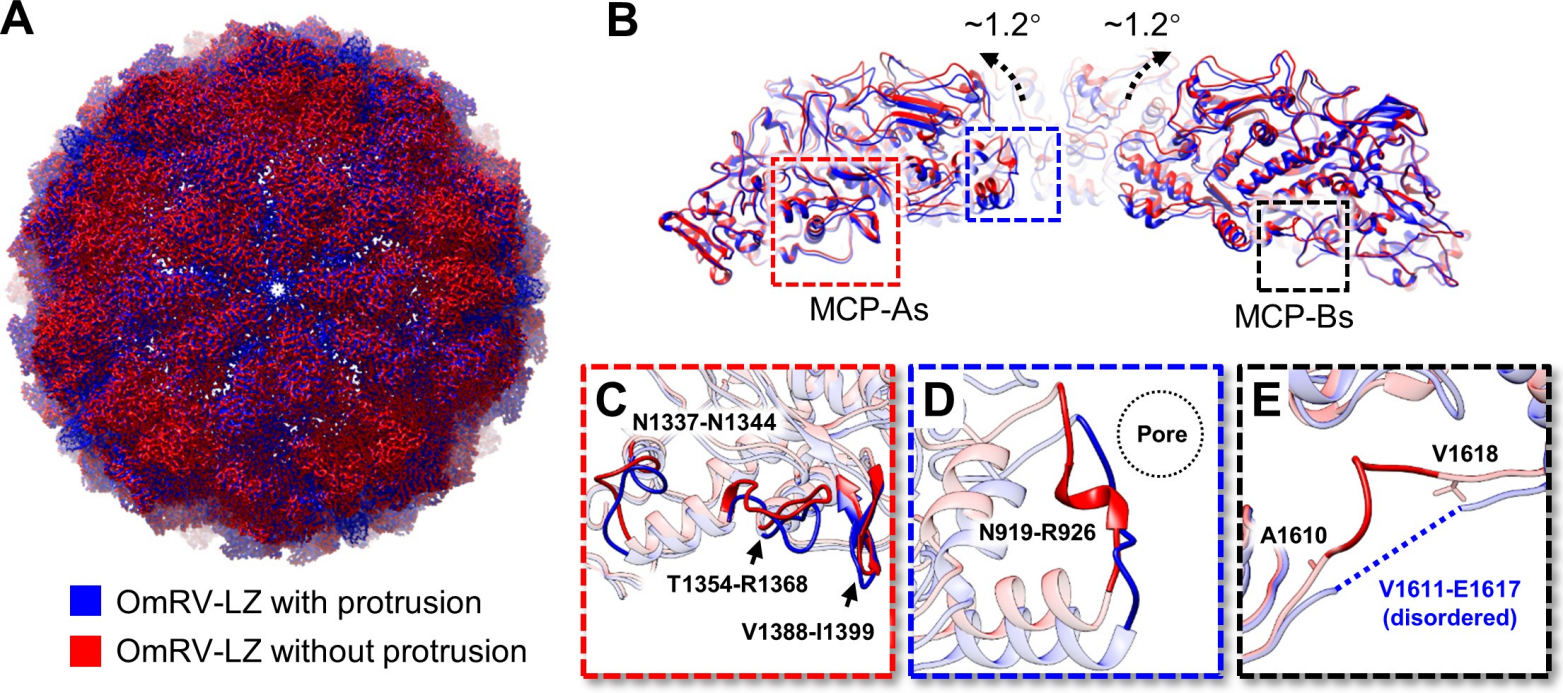
Structural comparison of OmRV-LZ capsid with and without the protrusion. **(A)** Superposition of the cryo-EM maps of the two capsids with (blue) and without the protrusion (red). **(B)** Superposed atomic models of these two major capsids near the five-fold vertex. The direction of the tilt is indicated by black arrows. **(C-E)** Zoomed-in views of the regions in panel B. The relative position of the pore at the five-fold vertex is indicated by the dotted circle in panel D. The disordered residues 1611–1617 of MCP-B in the structure with the protrusion are indicated by the dotted line in panel E.

Therefore, we applied 3-D classification to the OmRV-LZ full particles, and the map without the protrusion was distinguished and finally determined at 2.95 Å (S1A Fig). The result reveals that the particles without the protrusion account for approximately 20% among full particles. The maps demonstrate that the structure of the capsid without the protrusion is highly similar to that with the protrusion but indeed is slightly larger (Fig 5A and 5B and S1 Movie). The absence of the protrusion leads to the opening of the blocked pore on the top of the 5-fold vertex of the capsid.

We built the atomic model of MCPs of the protrusion-free structure and compared it with that of the structure with the protrusion. The results reveal that the MCP dimers in the protrusion-free structure tilt ~1.2° outward compared with those in the structure with the protrusion (Fig 5B and S1 Movie). The tilt centre is located approximately at the crossover point of the two-fold axis of the capsid shell. In addition, several local conformational changes were found (Fig 5C–5E and S1 Movie). First, conformational changes in loop 1337–1344, loop 1354–1368 and a strand-loop-strand motif (1388–1399) of MCP-A were found at the interface between MCP-A and MCP-B (Fig 5C). Second, the loop from Asn919 to Arg926 in MCP-A deviates from the five-fold vertex slightly, enlarging the average pore diameter by ~3 Å (Fig 5D). These changes make the gap between MCP-A and MCP-B slightly larger. Third, the disordered residues 1611–1617 in MCP-B in the map with the protrusion are well organized in the protrusion-free structure and thus can be traced (Fig 5E). These results demonstrate that the absence of the protrusion leads to global and local structural changes in the capsid.

There are two possible reasons for the absence of the protrusion. One is that the protrusion disassociates to carry out some unrecognized function in its life cycle. Another possible reason is that the protrusion may drop off during the process of purification due to the weak interactions between the protrusion and capsid.

### Anti-protrusion antibody affects the infectivity of OmRV-LZ

**Fig 6:**
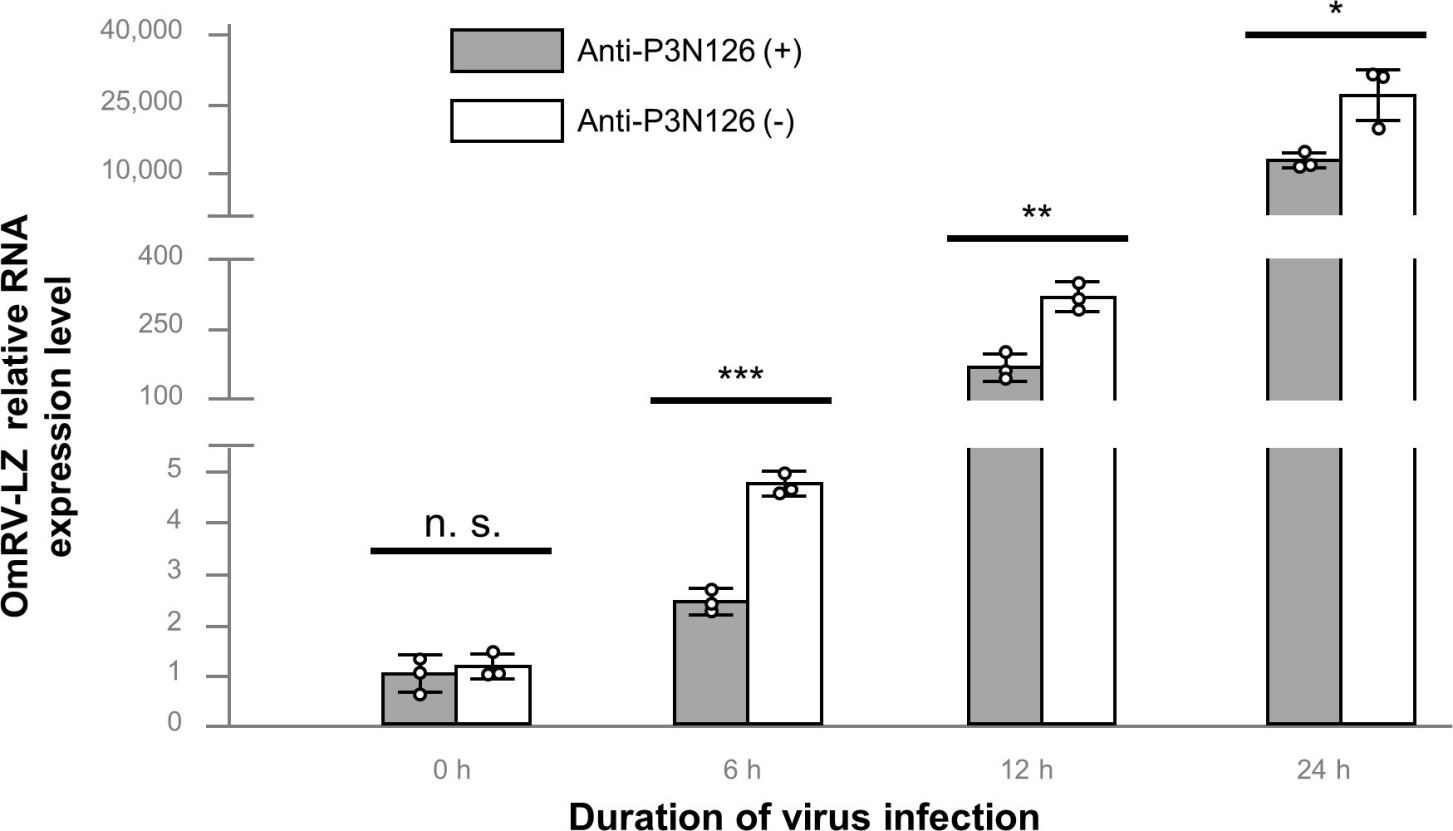
Results of qPCR analysis. qPCR analysis of the relative RNA expression level of OmRV-LZ in C6/36 cells treated with or without anti-P3N126. Data are shown as the mean ± standard deviation of three biological replicates. Each biological replicate comprised of three technical replicates (n. s., not signficant; *p<0.05; **p<0.01; ***p<0.001).

The P3 N-terminal 126-aa fragment (P3N126), which forms the protrusion based on the structure and model of protrusion, was expressed and purified *in vitro* to obtain the polyclonal antibody anti-P3N126 by immunizing the rabbit. Excessive anti-P3N126 was incubated with OmRV-LZ. However, CPEs occurred in both C6/36 groups infected by OmRV-LZ or OmRV-LZ+anti-P3N126. We further applied quantitative real-time PCR (qPCR) to compare the relative expression of viral RNA (Fig 6). One group of the viruses was incubated with anti-P3N126, and the other was treated with an equivalent amount of phosphate-buffered saline (PBS) as a control. RNA samples were collected after 0, 6, 12, and 24 h of infection. At 0 h, the levels of viral RNA were almost the same between these two groups, confirming that the quantities of the viruses used in the two groups were the same (Fig 6). Then, the levels of viral RNA in all the experimental groups decreased by approximately half compared with the control group at 6, 12, and 24 h (Fig 6). These results show that the anti-P3N126 antibodies do affect the infectivity of OmRV-LZ, although they cannot completely prevent the infection. However, the P-value between the two groups increased from 6 to 24 h of infection, indicating that anti-P3N126 affects the early stage of OmRV-LZ infection.

## Discussion

### The protrusion is implicated in the extracellular transmission of OmRV-LZ

The newly isolated mosquito-specific virus strain OmRV-LZ shares high homology in both sequence and structure with the previously reported OmRV-AK4 [7,22], except for the unexpected protrusion (Fig 2B). The protrusion structure is formed by N-terminal 126 residues of P3. The anti-P3N126 antibody reduced the infectivity of OmRV-LZ to C6/36 cells (Fig 6), suggesting that the antibody might block the attachment of the protrusion to the receptor, or might sterically obstruct the virus to approach the cell surface.

Undoubtedly, extracellular transmission primarily requires the ability to recognize, bind and enter a cell. The protrusion occupies the outermost capsid in the OmRV-LZ (Fig 2B). Therefore, the protrusion is most probably the first part attached to the cell during invasion. This is consistent with the findings in IMNV, which anchors the fibre-like protrusion on its capsid [18]. In addition to protrusions, extracellular transmission has also been found in both of IMNV and OmRV, the hosts (shrimp and mosquito) of which are metazoan [7,15]. To date, the structures of five assigned members of the fungus and protozoan virus family Totiviridae (ScV-L-A, *Helminthosporium victoriae* virus 190S, *Trichomonas vaginalis* virus 1, *Giardia lamblia virus* and *Leishmania* RNA viruses 1) have been determined, showing that these viruses lack a fibre-like protrusion on the capsid [23,26–29]. On the other hand, these viruses are also incapable of extracellular transmission, except for GLV [28]. Compared with viruses that infect fungi or protozoans, IMNV and OmRV may face a more complex environment when invading metazoan hosts, and the protrusion is probably an adaption to that environment.

The head part of the protrusion is mainly composed of a β-jelly roll structure (Fig 4B). The β-jelly roll structure is common in various viruses. Among members of Reoviridae, for example, the β-jelly roll structure has been found in P8 of rice dwarf virus [30], μ1 of orthoreovirus [31], and VP5 of grass carp reovirus [32]. In each case, hundreds of copies of these proteins form a layer of the T = 13 capsid shell and play an important role in cell invasion [30–32]. Both members of Totiviridae and Reoviridae belong to the dsRNA virus and have a relative smooth T = 1 capsid. With similar β-jelly roll structure, the protrusion of OmRV-LZ and the outer capsid proteins of members of Reoviridae, may have a similar function. The β-jelly roll structure has also been found in the receptor-binding head domain of fibre trimers in adenoviruses [33,34]. All of these clues suggest that the β-jelly roll in OmRV-LZ protrusion may also play roles in cell invasion.

However, the mechanism of extracellular transmission of OmRV-LZ needs to be further studied.

### Disassociation of the protrusion might be involved in different life stages of OmRV-LZ

The inner core of the protrusion is negatively charged (S3 Fig) and it might interact with positively charged substrate. OmRV-LZ may ingress into host cells by endocytosis. In the endocytosis vesicle’s low pH environment, the protrusions interact with the protons inside the pores, may induce conformation changes on the protrusions, thus causing breakage or disassociation.

The structure of the particles without protrusions reveals that disassociation of the protrusion leads to several conformational changes and slight expansion of capsid (Fig 5), which is reminiscent of the situation of a transcribing cypovirus (CPV) [35]. CPV, another dsRNA virus belonging to the family Reoviridae, loses its spike-like protein complexes (A-spikes) when it shifts to the transcribing state, and the shell becomes slightly enlarged [35].

On the other hand, the disassociation of the protrusions causes the pore at five-fold vertex expanding slightly, which would benefit the release of nascent single-stranded RNA, and therefore might trigger the transcription. However, more evidences and further investigations are needed.

### Comparison of the major capsid subunits of OmRV-LZ with that of other dsRNA viruses

The capsid protein subunit of OmRV-LZ shares a conserved core structure with that of ScV-L-A, containing five pairs of well-aligned α-helices (S2B Fig). Moreover, such conserved structure can also be found in capsid subunits of *Leishmania* RNA viruses 1 (Totiviridae) [29], *Penicillium chrysogenum* virus (Chrysoviridae) [36] and *Rosellinia necatrix* quadrivirus 1 (Quadriviridae) [37] indicating a preserved lineage of these dsRNA viruses. As expected, this close relationship is consistent with the phylogenetic analysis of the dsRNA viruses [38].

Commonly, MCP dimers form a capsid enwrapping the genome in many dsRNA viruses. In several members of Reoviridae, the N-terminus of subunit B of the inner capsid often forms a long arm interacting with other capsid subunits to enhance the stability of the capsid shell [30,39,40]. However, in OmRV-LZ, the C-terminus of MCP-B (Fig 3C), instead of N-terminus, play roles in stabilizing the capsid.

## Materials and methods

### Cell culture

C6/36 *Aedes albopictus* mosquito cells (ATCC, CRL-1660) were provided and maintained by the Guangdong Provincial Center for Disease Control and Prevention. The cells were cultured in complete medium containing 60% Dulbecco’s modified Eagle’s medium (Gibco, Grand Island, USA), 30% RPMI 1640 medium (Gibco), 10% foetal bovine serum (Gibco), and 100 U/mL penicillin/100 μg/mL streptomycin (Gibco) at 28°C with 5% CO_2_.

### Virus isolation, propagation and purification

*Culex* samples were collected from Leizhou, Guangdong Province (PR of China). Samples were mixed with complete medium, homogenized on Precellys 24 (Bertin Technologies SAS, Montigny-le-Bretonneux, France), and centrifuged at 10,000 × g for 30 min to remove the debris. The supernatants were added to C6/36 cells (1:100 v/v) in 24-well culture plates for 7 days. After three blind passages, the culture medium was used as a virus stock.

For virus purification, cell fluid was collected from at least four 175 cm^2^ cell culture flasks at 72 h p.i. The fluid was centrifuged at 10,000 × g for 30 min to remove the cell debris. Then, the supernatant was loaded onto a 30% sucrose cushion and centrifuged for 3 h at ~80,000 × g in an SW28 rotor (Beckman Coulter, Miami, USA) to aggregate the viruses. The pellets were resuspended in PBS and further centrifuged on a 30–50% cesium chloride (CsCl) gradient (~130,000 × g; SW41 rotor; 15 h). Two bands (upper and lower bands) were collected separately. To remove CsCl, each fraction was diluted in excess PBS and pelleted by centrifugation at ~130,000 × g for 3 h. The final pellets were recovered in ~100 μL of PBS.

### Viral genome extraction and sequencing

The total genome was extracted from a purified virus suspension (full particles) using the QIAamp MinElute Virus Spin Kit (Qiagen, Duesseldorf, Germany) according to the manufacturer’s instructions. cDNA was synthesized using the SuperScript IV First-Strand Synthesis System (Invitrogen, Carlsbad, USA) with random hexamers, according to the manufacturer’s instructions.

For next-generation sequencing of the viral genome, the TruSeq Stranded Total RNA Library Prep Kit (Illumina, San Diego, USA) was used for library preparation, and sequencing was performed on the HiSeq X Ten platform (Illumina). In total, 1 Gb of clean paired-end (150 bp) reads were quality trimmed by *Fastp* [41], and contigs were de novo assembled by *Megahit* [42]. Finally, the contig sequences were analysed using NCBI BLAST tools.

### Preparation of cryo-EM samples and data collection

Cryo-EM samples were prepared as previously described [43]. Briefly, newly glow discharged R1.2/1.3 holey copper grids (Quantifoil Micro Tools GmbH, Jena, Germany) were coated with a thin layer of freshly made continuous carbon film shortly before applying samples. Next, 2.5 μL of samples of purified full and empty viruses were applied to the grids, blotted, and flash-frozen in precooled liquid ethane using a Vitrobot Mark IV machine (Thermo Fisher Scientific, Waltham, USA) at 100% humidity.

Cryo-EM data of the full viruses were collected under a Titan Krios 300 kV electron microscope (Thermo Fisher Scientific) equipped with a Falcon III camera (Thermo Fisher Scientific) working in linear mode. Automatic data collection was performed with *EPU* software (Thermo Fisher Scientific) with defocus ranging from 1.0 μm to 3.0 μm. Movies were recorded with 39 frames under a dose rate of 1e^-^/Å^2^/s, giving a total dose of ~39 e^-^/Å^2^. The nominal magnification was 75,000× giving a calibrated pixel size of 1.09 Å.

Cryo-EM data of the empty viruses were collected under a Talos Arctica 200 kV electron microscope (Thermo Fisher Scientific) equipped with a K3 camera (Gatan, Pleasanton, USA) working in super-resolution mode. Automatic data collection was performed with *SerialEM* software [44] with defocus ranging from 1.0 μm to 2.5 μm. Movies were recorded with 34 frames under a dose rate of 37.33 e^-^/Å^2^/s, giving a total dose of ~60 e^-^/Å^2^. The nominal magnification was 54,000×, giving a calibrated pixel size of 0.9 Å. Statistics for data collection are summarized in S1 Table.

### Cryo-EM data processing

Both sets of cryo-EM data were processed in the same way as in the initial step. Beam-induced motion correction and dose weighting were performed using *MotionCor2* [45]. Contrast transfer function parameters were estimated using *Gctf* [46]. Virus particles were automatically selected by using program *Ethan* [47]. Several rounds of reference-free 2-D classification were performed using *Relion3*.*1* [48] to clean up the particles. A total of 57,261 full particles and 15,240 empty particles were finally selected for further data processing.

3-D classification was performed using *Relion3*.*1* [48] to distinguish the viruses with and without the protrusion. Then, the results were further refined and filtered using *JSPR* [49], generating the final reconstruction.

After the refinement of the whole virus, the head part of protrusion was further classified and refined. The coordinators of protrusions were estimated using shift and rotation parameters (shift x, y and three Euler angles) of the whole virus, which was obtained during previous refinement, by a modified script from a "block-based" reconstruction method [50]. Then, the protrusion sub-particles were extracted from the virus particles, and the capsid signal was subtracted in advance using *Relion3*.*1* [48]. The initial refinement parameter of the protrusion was inherited from the whole virus, except that the shift was set to 0 and the defocus was adjusted according to the distance from the protrusion to the particle plane. Several rounds of 3-D classification were performed by removing bad particles using *Relion3*.*1* [48]. The shift and rotation parameters of surviving particles were fine-tuned by local refinement to generate the final map.

The resolution of all the maps was estimated using the “gold-standard” FSC with the 0.143 criterion [51,52]. The maps were sharpened using *Relion3*.*1* and displayed and segmented using UCSF *Chimera* [53].

Some of the small proteins were found in the micrographs from the data of the empty viruses and were boxed manually. A total of 10,749 particles were boxed, and reference-free 2-D classification was performed. Due to the limited orientations, we did not conduct further refinement and 3-D reconstruction.

### Model building and structure analysis

The atomic models of MCP-A, MCP-B and the protrusion were built in *COOT* [54] based on the electron density map of the full virus with the protrusion and were refined in *Phenix* [55].

The qualities of all the atomic models were validated by using *Phenix* [55]. The surface electrostatic potential of the structures was calculated using *APBS* [56]. Statistics for refinement and validation data are summarized in S1 Table.

### Antibody

A pair of primers was designed to clone the gene of the ORF1 P3 N-terminal 126-aa fragment (P3N126):

Forward: ATATATGGATCCATCGACTGCGACTCATCA;

Reverse: ATATATCTCGAGAGCTGGTGCGTAAGGAGT.

Amplified PCR products were ligated into the pET28a plasmid, and the recombinant plasmids were transformed into *E*. *coli* DH5α and *E*. *coli* BL21 (DE3) for amplification and expression, respectively. Purification of recombinant proteins with His-tags was performed by using a Ni-NTA affinity column. Purified recombinant proteins were used to immunize New Zealand rabbits to produce serum and anti-P3N126 antibodies, which were made by GenScript Company (Nanjing, China). The specificity and the range of reactivity of the antibodies have been validated by the manufacturer.

### Quantitative real-time PCR

To test the effect of the anti-P3N126 fragment antibody on the infectivity of OmRV-LZ, aliquots of OmRV-LZ viruses were incubated with antibodies and an equal volume of PBS (negative control) at 4°C overnight. After incubation, the viruses were added with monolayer C6/36 cells in 12-well culture plates. Infection was stopped by adding TRIzol reagent (Invitrogen) onto the cells at 0, 6, 12 and 24 h p.i.. Total RNA was extracted according to the manufacturer’s instructions. qPCR was performed using the StarScript II Green Fast One-Step qRT-PCR Kit (Genstar, Beijing, China) in a Lightcycler 480 system (Roche, Basel, Switzerland). Relative gene expression was analysed according to the Livak 2^*-ΔΔCt*^ method [57]. Statistical significance of the mean value was determined by the independent-sample T-test. Specific primers designed from the OmRV-LZ genome and for the *A*. *albopictus* β-actin gene used in qPCR are listed below [58]:

OmRV-LZ-Forward: AACGTCTGTGGCCATCTCTG

OmRV-LZ-Reverse: CAGCAGCTCTTTGCGTGTTT

*Aedes albopictus* β-actin-Forward: TGACTGAACGTGGCTACTCG

*Aedes albopictus* β-actin-Reverse: ACTTCTCGAGGGAGGAGGAC

## Supporting information

S1 Fig Qualities of the cryo-EM maps. **(A)** Fourier shell curves of the OmRV-LZ full particle with the protrusion (2.79 Å), full particle without the protrusion (2.95 Å), empty particle with the protrusion (3.40 Å) and the protrusion structure obtained from C5 reconstruction (4.10 Å). **(B)** Representative atomic models and the corresponding cryo-EM maps of the α-helix and several residues with large sidechains.
(TIF)

S2 Fig Structural comparison between the major capsid proteins of OmRV-LZ and ScV-L-A. **(A)** Superposition of MCP-A in OmRV-LZ (blue) and ScV-L-A (pink). **(B)** Zoomed-in view of the region framed by the yellow dotted box in panel B, showing five pairs of α-helices (labelled α1–5) with conserved folds between OmRV-LZ and ScV-L-A. Other mismatched structures are set to translucent for clarity. **(C, D)** Zoomed-in view of the region framed by the green dotted box in panel B. **(C)** Atomic model of the cap-snatching reaction centre in ScV-L-A. The sidechain of the active site His154 (coloured pink) and nearby key residues Tyr150, His151, Asp152, Tyr452, Tyr538 and Asp540 that may be involved in the cap-snatching reaction (coloured grey) are displayed. **(D)** Atomic model of the region in OmRV-LZ MCP-A corresponding to the cap-snatching reaction centre of ScV-L-A MCP-A. All sidechains in this region are displayed.
(TIF)

S3 Fig Electrostatic potential analyses of the protrusion. **(A)** Protrusion structure surface rendered with electrostatic potentials. **(B)** The sidechain of Tyr367 of the protrusion is inserted into the positively charged canyon area composed of two neighbouring MCP-As that are superposed with the ribbon model and rendered with electrostatic potentials.
(TIF)

S4 Fig 2-D Classification of the OmRV-LZ full particles. Reference-free 2-D classification of the OmRV-LZ full particles. Two classes without the protrusion are highlighted by red boxes.
(TIF)

S1 Movie Conformational changes between the two states of OmRV-LZ. Model morph of capsid decamer and MCP-A monomer between OmRV-LZ structure with protrusion (blue) and without protrusion (red), showing the global tilt and local conformational changes.
(MP4)

S1 Table Summary of data collection, refinement and validation statistics. (DOCX)
