# Supplementary figures and images for: Cryo-EM reveals a previously unrecognized structural protein of a dsRNA virus implicated in its extracellular transmission
Source: PLoS Pathog. 2021 Mar 17;17(3):e1009396. doi: 10.1371/journal.ppat.1009396 (PMC7968656; doi:10.1371/journal.ppat.1009396)

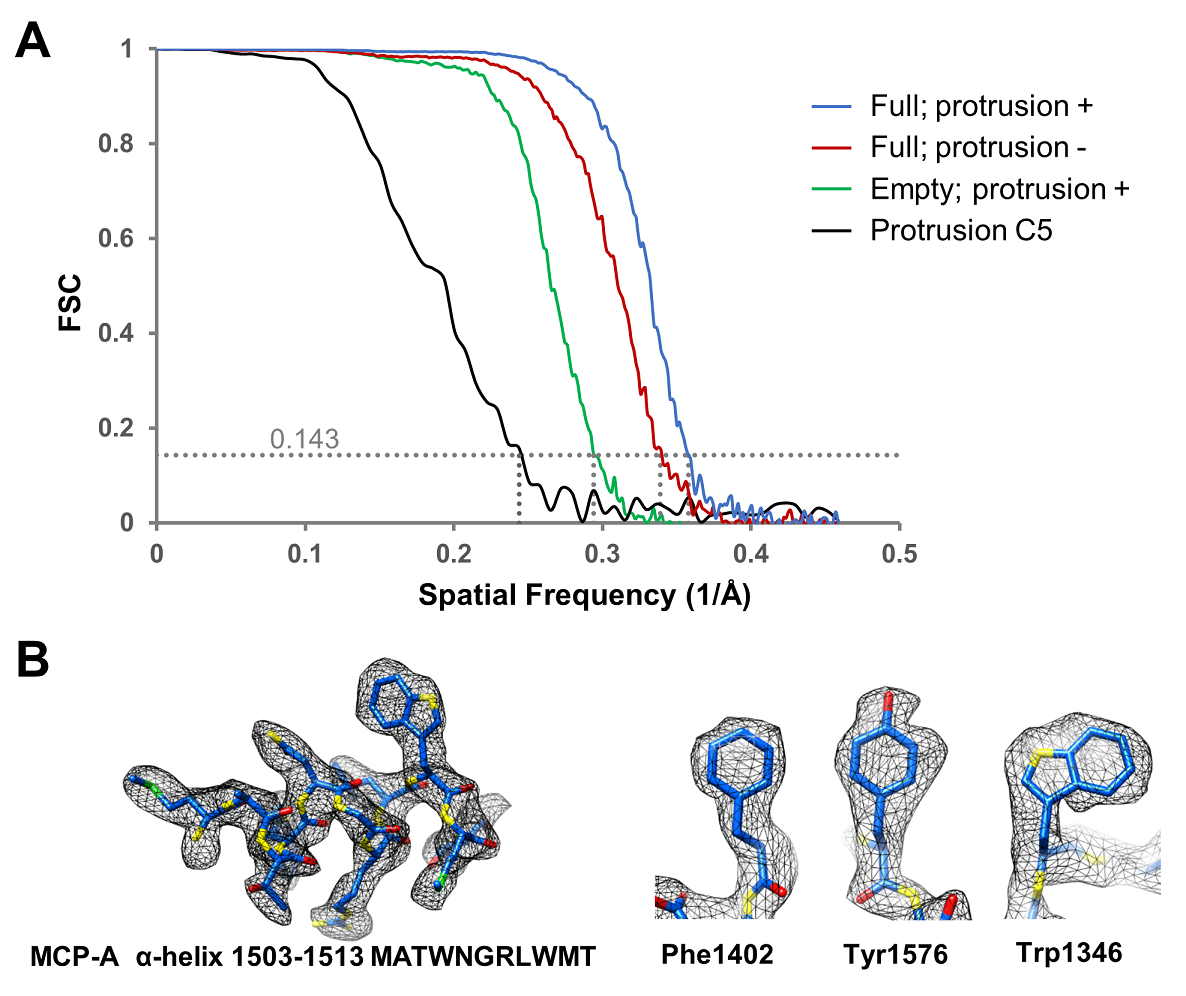

Supplement: S1 Fig — (A) Fourier shell curves of the OmRV-LZ full particle with the protrusion (2.79 Å), full particle without the protrusion (2.95 Å), empty particle with the protrusion (3.40 Å) and the protrusion structure obtained from C5 reconstruction (4.10 Å). (B) Representative atomic models and the corresponding cryo-EM maps of the α-helix and several residues with large sidechains. (TIF) [file ppat.1009396.s001.tif]

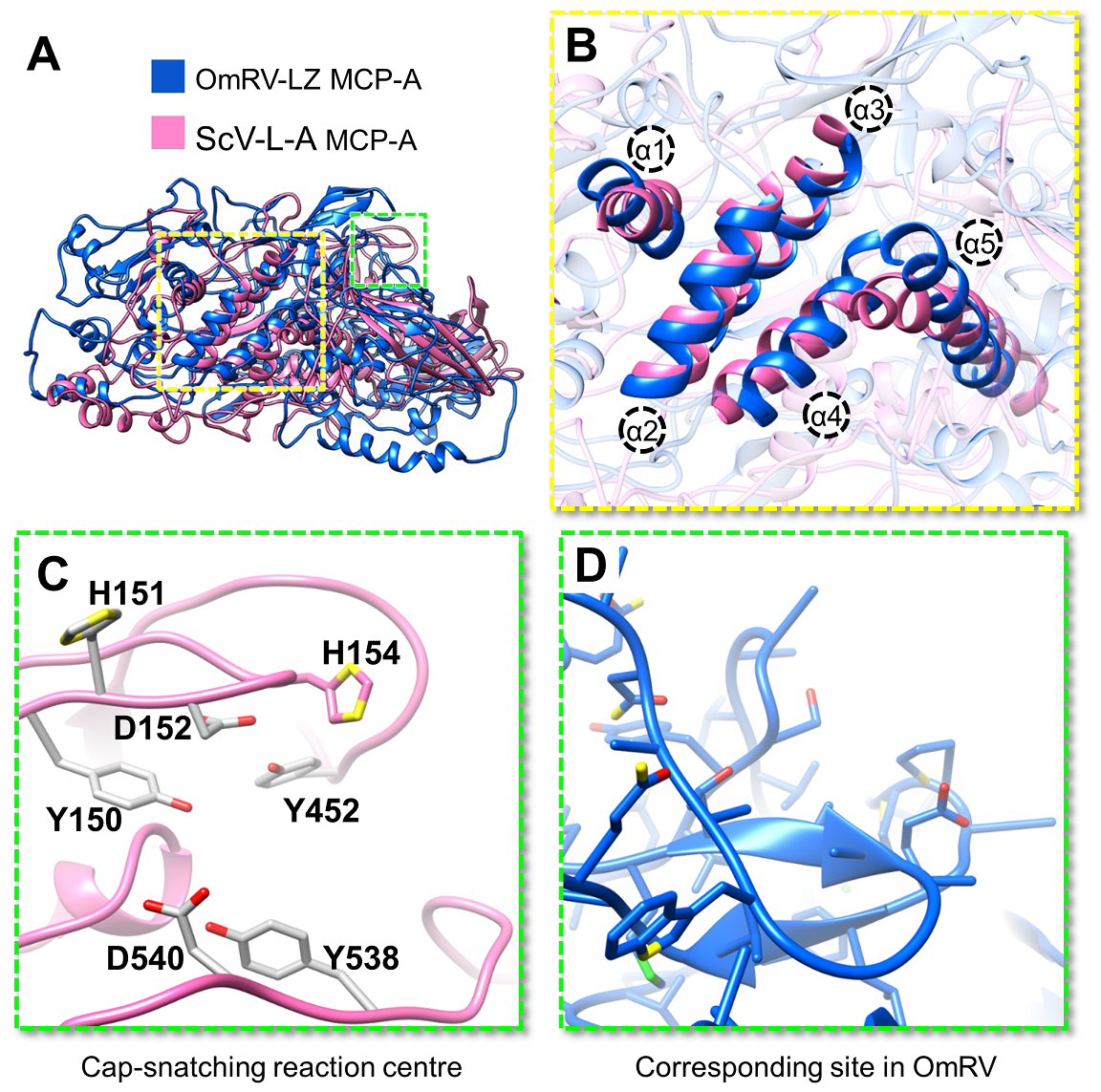

Supplement: S2 Fig — (A) Superposition of MCP-A in OmRV-LZ (blue) and ScV-L-A (pink). (B) Zoomed-in view of the region framed by the yellow dotted box in panel B, showing five pairs of α-helices (labelled α1–5) with conserved folds between OmRV-LZ and ScV-L-A. Other mismatched structures are set to translucent for clarity. (C, D) Zoomed-in view of the region framed by the green dotted box in panel B. (C) Atomic model of the cap-snatching reaction centre in ScV-L-A. The sidechain of the active site His154 (coloured pink) and nearby key residues Tyr150, His151, Asp152, Tyr452, Tyr538 and Asp540 that may be involved in the cap-snatching reaction (coloured grey) are displayed. (D) Atomic model of the region in OmRV-LZ MCP-A corresponding to the cap-snatching reaction centre of ScV-L-A MCP-A. All sidechains in this region are displayed. (TIF) [file ppat.1009396.s002.tif]

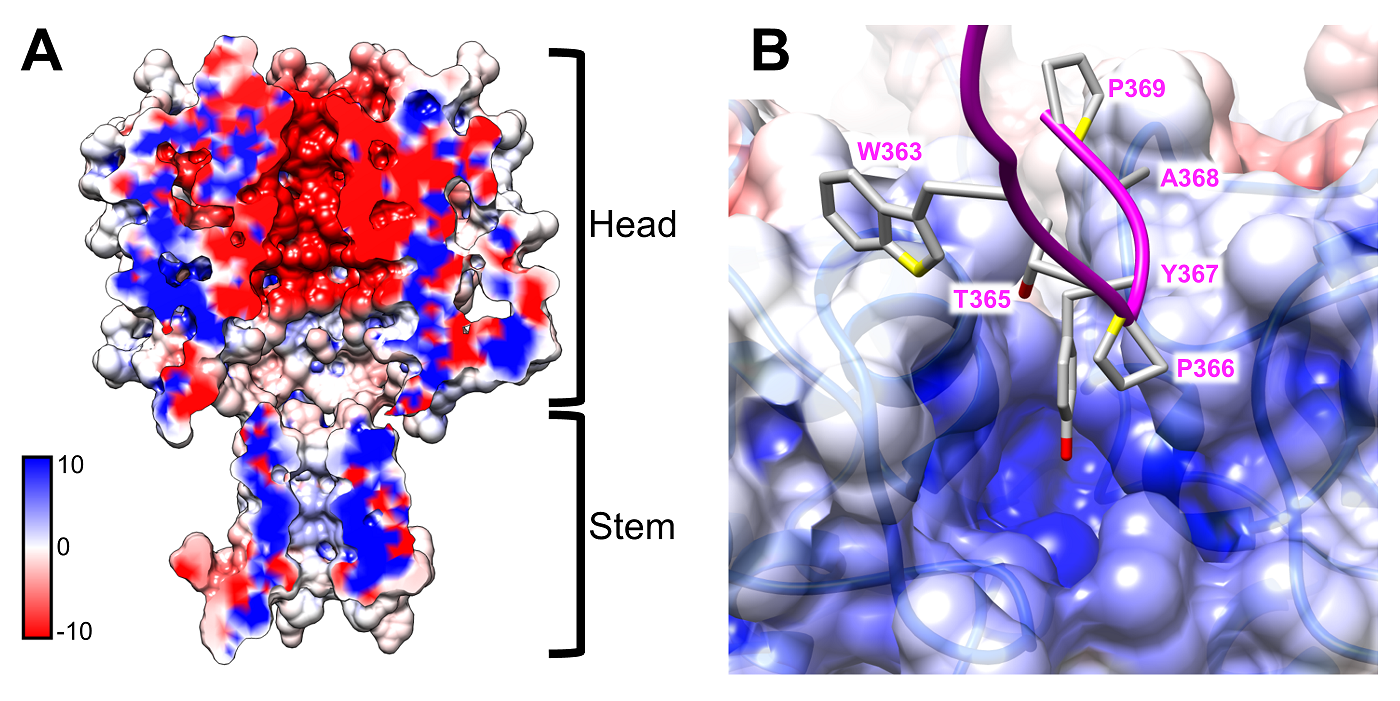

Supplement: S3 Fig — (A) Protrusion structure surface rendered with electrostatic potentials. (B) The sidechain of Tyr367 of the protrusion is inserted into the positively charged canyon area composed of two neighbouring MCP-As that are superposed with the ribbon model and rendered with electrostatic potentials. (TIF) [file ppat.1009396.s003.tif]

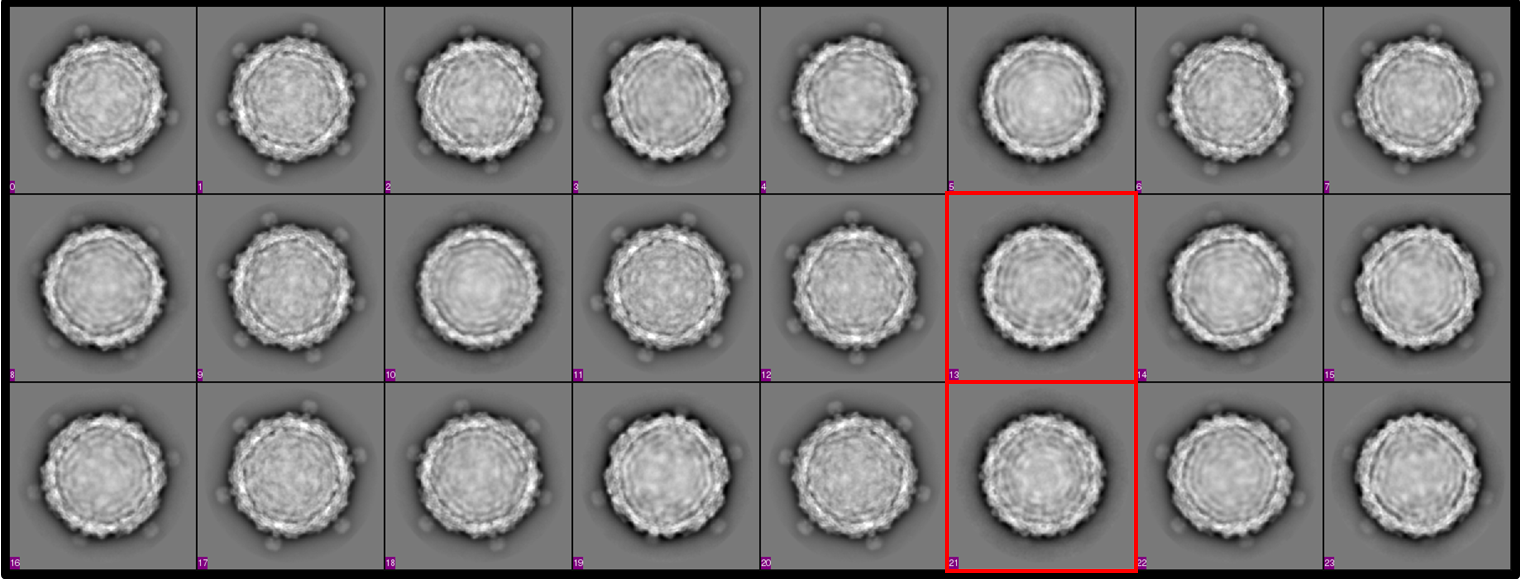

Supplement: S4 Fig — Reference-free 2-D classification of the OmRV-LZ full particles. Two classes without the protrusion are highlighted by red boxes. (TIF) [file ppat.1009396.s004.tif]
