# Supplementary material for: Cryo-EM reveals a previously unrecognized structural protein of a dsRNA virus implicated in its extracellular transmission
Source: PLoS Pathog. 2021 Mar 17;17(3):e1009396. doi: 10.1371/journal.ppat.1009396 (PMC7968656; doi:10.1371/journal.ppat.1009396)
Supplement: S1 Table — (DOCX) [file ppat.1009396.s006.docx]

S1 Table. Summary of data collection, refinement and validation statistics.

|  | OmRV-LZ full particle with protrusion | OmRV-LZ full particle without protrusion | OmRV-LZ Protrusion | OmRV-LZ empty particle |
| --- | --- | --- | --- | --- |
|  | EMD-30537  PDB 7D0K | EMD-30538  PDB 7D0L | EMD-30507 PDB 7CZ6 | EMD-30539 |
| **Data collection and processing** | | | | |
| Magnification | 75,000 × | 75,000 × | 75,000 × | 54,000 × |
| Voltage (kV) | 300 | 300 | 300 | 200 |
| Electron exposure (e-/Å^2^) | ~39 | ~39 | ~39 | ~60 |
| Defocus range (μm) | 1.0-3.0 | 1.0-3.0 | 1.0-3.0 | 1.0-2.5 |
| Pixel size (Å) | 1.09 | 1.09 | 1.09 | 0.90 |
| Symmetry imposed | I | I | C5 | I |
| Initial particle images (no.) | 76,348 | 20,320 | 1,160,016 | 19,120 |
| Final particle images (no.) | 57,261 | 15,240 | 38,935 | 12,483 |
| Map resolution (Å) | 2.79 | 2.95 | 4.10 | 3.40 |
| FSC threshold | 0.143 | 0.143 | 0.143 | 0.143 |
| **Refinement and validation** | | | | |
| Map sharpening B factor (Å^2^) | -175.35 | -174.96 | -114.97 | -135.77 |
| Model composition |  | | | |
| Non-hydrogen atoms | 13,253 | 13,304 | 907 | - |
| Protein residues | 1,739 | 1,746 | 126 | - |
| R. m. s. deviations |  | | | |
| Bond lengths (Å) | 0.009 | 0.011 | 0.008 |  |
| Bond angles (°) | 1.22 | 1.27 | 1.30 | - |
| MolProbity score | 1.71 | 1.88 | 2.15 | - |
| Clashscore | 9.83 | 11.92 | 14.36 | - |
| Poor rotamers (%) | 0 | 0 | 0 | - |
| Ramachandran plot |  | | | |
| Favored (%) | 96.83 | 95.87 | 91.94 | - |
| Allowed (%) | 3.17 | 4.13 | 8.06 | - |
| Disallowed (%) | 0 | 0 | 0 | - |
| Map CC (main chain/side chain) | 0.82/0.79 | 0.84/0.82 | 0.54/0.54 | - |
| d99 | 2.2 | 3.1 | 4.0 | - |
